# Central Administration of Galanin Receptor 1 Agonist Boosted Insulin Sensitivity in Adipose Cells of Diabetic Rats

**DOI:** 10.1155/2016/9095648

**Published:** 2016-04-05

**Authors:** Zhenwen Zhang, Penghua Fang, Biao He, Lili Guo, Johan Runesson, Ülo Langel, Mingyi Shi, Yan Zhu, Ping Bo

**Affiliations:** ^1^Department of Endocrinology, Clinical Medical College, Yangzhou University, Yangzhou, Jiangsu 225001, China; ^2^Department of Physiology, School of Hanlin, Nanjing University of Chinese Medicine, Taizhou, Jiangsu 225300, China; ^3^Key Laboratory of Gerontology, Medical College, Yangzhou University, Yangzhou, Jiangsu 225001, China; ^4^Department of Neurochemistry, Arrhenius Laboratories for Natural Sciences Stockholm University, 10691 Stockholm, Sweden

## Abstract

Our previous studies testified the beneficial effect of central galanin on insulin sensitivity of type 2 diabetic rats. The aim of the study was further to investigate whether central M617, a galanin receptor 1 agonist, can benefit insulin sensitivity. The effects of intracerebroventricular administration of M617 on insulin sensitivity and insulin signaling were evaluated in adipose tissues of type 2 diabetic rats. The results showed that central injection of M617 significantly increased plasma adiponectin contents, glucose infusion rates in hyperinsulinemic-euglycemic clamp tests, GLUT4 mRNA expression levels, GLUT4 contents in plasma membranes, and total cell membranes of the adipose cells but reduced the plasma C-reactive protein concentration in nondiabetic and diabetic rats. The ratios of GLUT4 contents were higher in plasma membranes to total cell membranes in both nondiabetic and diabetic M617 groups than each control. In addition, the central administration of M617 enhanced the ratios of pAkt/Akt and pAS160/AS160, but not phosphorylative cAMP response element-binding protein (pCREB)/CREB in the adipose cells of nondiabetic and diabetic rats. These results suggest that excitation of central galanin receptor 1 facilitates insulin sensitivity via activation of the Akt/AS160 signaling pathway in the fat cells of type 2 diabetic rats.

## 1. Introduction

The growing evidence supports the fact that neuropeptide galanin (GAL) can regulate energy homeostasis and insulin sensitivity of animals. The high densities of GAL-immunoreactivity are found in the hypothalamus [1], which is an important center in controlling energy metabolism [2]. An injection of GAL into paraventricular nucleus (PVN) significantly increased caloric intake and body weight of rats, as well as circulating nonesterified fatty acid levels and lipoprotein lipase activity in adipose tissue [3]. GAL-knockout mice showed impaired glucose disposal due to reduced insulin response and insulin-independent glucose elimination [4] and consumed less energy and lost more body weight compared to controls on a high-fat diet [5], whereas in homozygous GAL-transgenic mice the metabolic rates of lipid and carbohydrate were increased due to improved insulin sensitivity [6]. In addition, the results of our and others' studies revealed that abdominal or intracerebroventricular injection (i.c.v.) of M35, a galanin antagonist, significantly increased insulin resistance and inhibited the glucose transporter 4 (GLUT4) translocation from intracellular membrane compartments to cell surfaces in skeletal muscle and fat tissue of rats [7–12].

All of three GAL subtype receptors (GalR1-3) are G-protein-coupled isoforms. ^25^I-GAL binding assay showed that GalR1 accounted for approximately 90% of all GAL binding sites in the PVN [13]. The GalR1 mRNA is abundantly expressed in many brain regions, including hypothalamus and forebrain regions [14]. Its expression level, not GalR2 mRNA or GalR3 mRNA, is positively correlated with the GAL content in the hypothalamic nuclei of GAL-transgenic mice [14, 15]. But so far it is unclear whether central GalR1 mediates the beneficial effect of GAL on insulin sensitivity in fat tissues of animals. Therefore, in this study the effects of i.c.v. injection of a GalR1 agonist, M617 on insulin sensitivity, and insulin signaling in the adipose tissue of type 2 diabetic rats were evaluated. Because GalR1 antagonist cannot be found anywhere, we cannot survey the role of the antagonist in the current experiment.

## 2. Experimental Procedures

### 2.1. Drugs and Reagents

M617 was synthesized using Fmoc chemistry. The synthesis and final cleavage from the resin using Fmoc chemistry were carried out as described by Sollenberg et al. [16]. Streptozotocin was obtained from Sigma-Aldrich Inc., USA. Antibodies against GLUT4, pAkt, Akt, pAS160, AS160, phosphorylation of cAMP response element-binding protein (pCREB), and CREB were acquired from Santa Cruz Biotechnology Inc., USA. Trizol reagent was obtained from Gibco Invitrogen, USA. Rat C-reactive protein (CRP) and adiponectin ELISA kits were obtained from Uscn Life Science, Inc. Wuhan, China.

### 2.2. Diabetic Models

Seventy-six 250 ± 10 g male Wistar rats, coming from Yangzhou University Animal Center, were kept in the polypropylene cages and under the conditions of temperature 21 ± 2°C, humidity 54%, 12-hour light-dark cycles, high-fat diet (59% fat, 21% protein, and 20% carbohydrate), and water ad libitum. After eight weeks, forty-four of the animals were treated with streptozotocin (35 mg/kg i.p.) in 0.1 mM citrate buffer (pH 4.5) under a fasted state. The tail blood of the rats was weekly taken to determine the blood glucose levels with a Glucometer (HMD Biomedical, Taiwan) during the study. After being fed with the high-fat diet for another four weeks, thirty-two animals with fasting glucose concentration over 11.1 mmol/L as well as polydipsia, polyphagia, polyuria, shaggy, cachexia, and weight loss were taken as models of diabetes [10, 17]. The diabetic rats were randomly distributed into diabetic control (*n* = 16) and diabetic M617 (*n* = 16) groups. In addition, thirty-two nondiabetic rats were evenly attributed to nondiabetic control and nondiabetic M617 groups. The rats in all groups were fed with the high-fat diet during the experiment. All animal procedures used were performed in accordance with the Guiding Principles for Care and Use of Experimental Animals. The experiments were approved by the Animal Studies Committee of Yangzhou University.

### 2.3. Intracerebroventricular Injection and Sample Collection

The method of i.c.v. injection is similar to those described previously in animals [10]. In brief, animals were anesthetized with 3% amobarbital sodium (50 mg/kg i.p.) and stereotaxically implanted with a guide cannula into the lateral ventricle: anterior-posterior (AP), −0.8 mm; L, 1.4 mm; and V, 3.3 mm. The cannula was cemented to four jeweler's screws attached to the skull and closed with an obturator. Its location was judged by the flow of cerebrospinal fluid. After recovery for 7 d from surgery, rats in the M617 group were injected with 1 nmol/kg M617 in 5 *μ*L artificial cerebrospinal fluid (in mM: 133.3 NaCl, 3.4 KCl, 1.3 CaCl_2_, 1.2 MgCl_2_, 0.6 NaH_2_PO_4_, 32.0 NaHCO_3_, and 3.4 glucose, pH 7.4 by 0.5 M hydrochloric acid) at 9:00 am every day for 21 days and rats in both control groups with 5 *μ*L vehicle. At the end of the experiment, half of rats in every group (*n* = 8) were anesthetized as above after fast 12 h. Then 4 mL artery blood and 5 g epididymal fat pad were collected. The blood was centrifugated at 3500 r.p.m. for 10 min to obtain the plasma. The plasma and fat pad were frozen at −80°C.

### 2.4. Hyperinsulinemic-Euglycemic Clamp Tests

In the hyperglycemic clamp tests, the other half of rats in every group (*n* = 8) was anesthetized and catheterized in right carotid artery and left jugular vein after fasted 12 h as previously described [7, 9]. The animals were infused with insulin at a constant rate of 2 mU/kg·min into the jugular vein until the end of the test. 10% glucose was infused at variable rates as needed to clamp glucose levels at 5 ± 0.5 mmol/L. The glucose infusion rates were calculated corresponding to the last 6 samplings at the clamp level. Once above experiments were completed, each rat was euthanized by infusion of amobarbital sodium and saturated potassium chloride. Their brains were checked to confirm the correct implantation of the cannulas.

### 2.5. Measurement of Plasma Adiponectin and CRP Levels

The adiponectin and CRP levels were quantified using each competitive ELISA kit.

### 2.6. Real-Time PCR

To determine the GLUT4 mRNA level, the total RNA from 100 mg of the frozen adipose tissue was isolated by Trizol according to the manufacturer's instructions. The concentration of the RNA was calculated by spectrophotometric assays of 260/280 nm, and the integrity of the RNA was assessed by running samples on a 1% formaldehyde agarose gel in TAE buffer (40 mmol/L tris-acetic acid, 1 mmol/L EDTA). cDNA was synthesized from 1 *μ*g RNA using MMLV reverse transcriptase.

The mRNA expression levels were determined using real-time fluorescent detection in an ExicyclerTM 96 PCR machine (LG Company, Korea). The oligonucleotide primers were as follows: GLUT4 forward 5′-ACAGGGCAAGGATGGTAGA-3′, reverse 5′-TGGAGGGGAACAAGAAAGT-3′, *β*-actin forward 5′-GGCTGTGTTGTCCCTGTATG-3′, reverse 5′-AATGTCACGCACGATTTCC-3′. Amplification condition was as follows: an initial denaturation at 95°C for 10 min; 95°C for 15 s; 62°C for 60 s; 40 cycles. The 2^−ΔCT^ method was used to analyze the real-time PCR data. Results were normalized with reference to *β*-actin [9].

### 2.7. Subcellular Fractionation of Adipose Cells

Membranes of fat cells were separated as described previously [9]. Briefly, fat pads were washed, homogenized in an ice-cold homogenization buffer (250 mmol/L sucrose, 2 mmol/L EDTA, 2.5 mmol/L Tris-HCl, 10 *μ*g/mL aprotinin, 10 *μ*g/mL leupeptin, and 100 *μ*mol/L phenylmethylsulfonyl fluoride, pH 7.4), and then centrifuged at 13,000 g for 20 min at 4°C to remove the fat cake. The infranatant was centrifuged at 31,000 g for 1 h to yield the low-density intracellular membranes. The pellet from the first spin was layered over a sucrose cushion and centrifuged at 75,000 g for 1 h. The interphase was removed and spun at 39,000 g for 20 min to yield the plasma membranes.

### 2.8. Western Blot Analysis

Western blot analyses were used to determine GLUT4, pAKT, Akt, pAS160, AS160, and pCREB and CREB levels in the adipose tissue. Briefly, fifty micrograms of samples was separated via electrophoresis on a 12% sodium dodecyl sulfate-polyacrylamide gel as described before [10]. The separated proteins were transferred to polyvinylidene difluoride filter membranes. Membranes were blocked in Tris-buffered saline (pH 7.5) containing 0.05% Tween-20 (1 × TBST) and 5% skimmed milk for 2 h and then probed with an antibody overnight at 4°C. Membranes were washed three times with 1 × TBST for 10 minutes and incubated for 2 h with horseradish peroxidase-conjugated secondary antibody. Lastly, immunoreactive bands were visualized by chemiluminescence and quantified by densitometry using a Quantity One Analysis Software (Bio-Rad). The *β*-actin levels were taken as the internal control. The sum of the GLUT4 concentration in intracellular membranes and in plasma membranes was calculated as the concentration of total cell membranes.

### 2.9. Statistical Analysis

Comparisons among the groups were analyzed by two-way ANOVA followed by Tukey's multiple-range tests. Data about food intake, body weight, plasma insulin, and free fatty acid levels before and after the experiments were compared by paired Student's *t*-test. Data were presented as mean ± SEM with *P* < 0.05 as the limit for statistical significance.

## 3. Results

### 3.1. Food Intake, Body Weight, Insulin, and Plasma Free Fatty Acid Levels

As showed in Table 1, central administration of M617 enhanced food intake and body weight by 9.0% (*P* < 0.05) and 5.3% (*P* < 0.05) in the diabetic M617 group compared with diabetic controls, also by 10.5% (*P* < 0.05) and 4.6% (*P* < 0.05) in the nondiabetic M617 group compared with nondiabetic controls, respectively. Both indexes after the experiments compared with before were augmented by 13.8% (*P* < 0.01) and 9.8% (*P* < 0.05) in the diabetic M617 group and by 13.5% (*P* < 0.01) and 9.1% (*P* < 0.05) in the nondiabetic M617 group, while the administration of M617 attenuated plasma insulin and free fatty acid levels by 12.1% (*P* < 0.05) and 38.7% (*P* < 0.01) in the diabetic M617 group compared with diabetic controls. Compared with nondiabetic controls, the plasma insulin levels were reduced by 16.0% (*P* < 0.05), while the free fatty acid levels were nonsignificantly decreased by 7.2% (*P* > 0.05) in the nondiabetic M617 group. Both plasma insulin and free fatty acid levels after the experiments compared with those before were decreased by 11.1% (*P* < 0.01) and 36.1% (*P* < 0.01) in the diabetic M617 group and by 19.4% (*P* < 0.01) and 11.2% (*P* < 0.05) in the nondiabetic M617 group, respectively.

### 3.2. Hyperinsulinemic-Euglycemic Clamping

**Figure 1:**
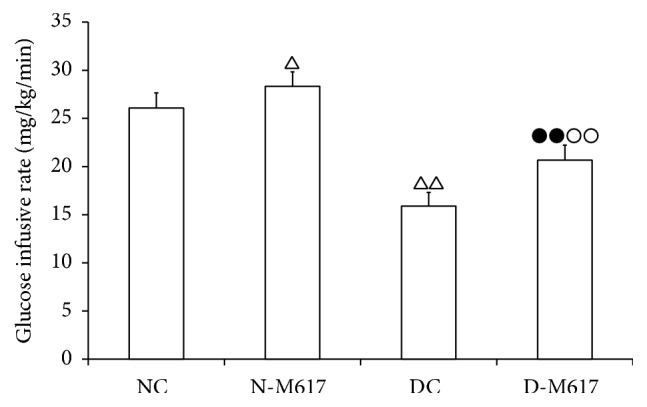
The i.c.v. administration of M617 enhanced the glucose infusing rates in hyperinsulinemic-euglycemic clamp tests (*n* = 8). The glucose infusing rates were higher in diabetic M617 (D-M617) and nondiabetic M617 (N-M617) groups compared with diabetic controls (DC) and nondiabetic controls (NC), respectively. The rates were lower in D-M617 and DC compared with N-M617 and NC, respectively. All data shown are the means ± SEM. ^△^ *P* < 0.05, ^△△^ *P* < 0.01 versus NC; ^●●^ *P* < 0.01 versus N-M617; ^○○^ *P* < 0.01 versus DC.

During the clamp tests, the glucose infusion rates were markedly elevated by i.c.v. injection with M617 (*F*[3,32] = 90.1, *P* < 0.0001).  Figure 1 showed that the infusion rate in the diabetic M617 group was increased by 30.2% (*P* < 0.01) compared with diabetic controls but reduced by 27.1% (*P* < 0.01) compared with the nondiabetic M617 group. Compared with nondiabetic controls, the rate was increased by 8.8% (*P* < 0.05) in the nondiabetic M617 group but attenuated by 39.1% (*P* < 0.01) in the diabetic control group.

### 3.3. Plasma CRP Levels

**Figure 2:**
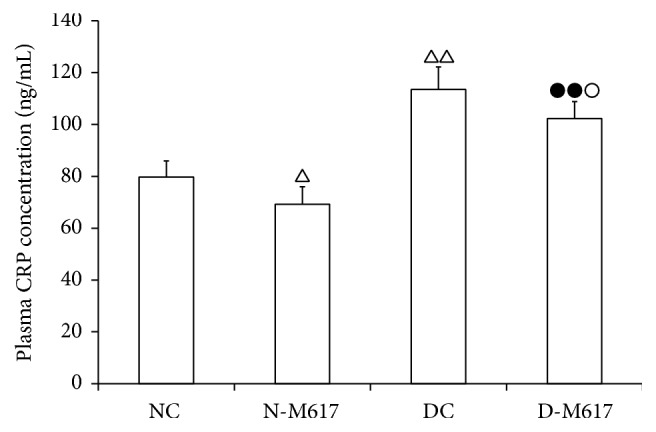
The changes of plasma C-reactive protein (CRP) concentration after i.c.v. injection of M617 in rats (*n* = 8). The plasma CRP contents were lower in diabetic M617 (D-M617) and nondiabetic M617 (N-M617) groups compared with diabetic controls (DC) and nondiabetic controls (NC), respectively. The CRP contents were higher in D-M617 and DC groups compared with N-M617 and NC groups, respectively. All data shown are the means ± SEM. ^△^ *P* < 0.05, ^△△^ *P* < 0.01 versus NC; ^●●^ *P* < 0.01 versus N-M617; ^○^ *P* < 0.05 versus DC.

To determine the effect of activation of central GalR1 on CRP liberation, we contrasted the plasma CRP concentration in the M617 group with corresponding controls. As shown in Figure 2, the i.c.v. injection of M617 reduced the CRP concentration (*F*[3,32] = 62.3, *P* < 0.001). The CRP concentration in the diabetic M617 group was decreased by 9.9% (*P* < 0.05) compared with diabetic controls but was elevated by 47.8% (*P* < 0.01) compared with the nondiabetic M617 group. Compared with nondiabetic controls, the concentration was decreased by 13.2% (*P* < 0.05) in the nondiabetic M617 group but increased by 42.4% (*P* < 0.01) in the diabetic control group. The results suggest that activation of GalR1 in brain may inhibit CRP secretion to ameliorate insulin resistance.

### 3.4. Plasma Adiponectin Concentration

**Figure 3:**
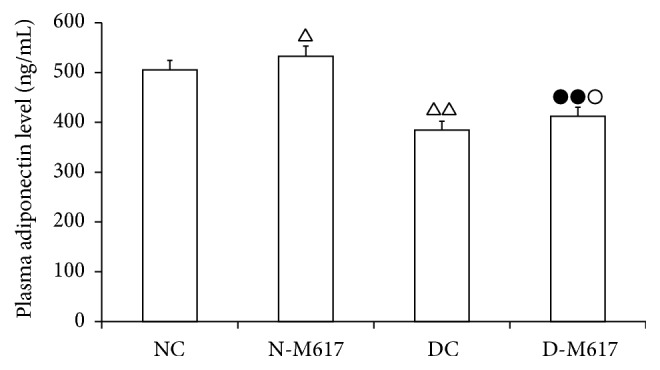
The alteration of plasma adiponectin concentration after i.c.v. injection of M617 in rats (*n* = 8). The plasma adiponectin concentration was higher in diabetic M617 (D-M617) and nondiabetic M617 (N-M617) groups compared with diabetic controls (DC) and nondiabetic controls (NC), respectively. The adiponectin contents were lower in D-M617 and DC groups compared with N-M617 and NC groups, respectively. All data shown are the means ± SEM. ^△^ *P* < 0.05, ^△△^ *P* < 0.01 versus NC; ^●●^ *P* < 0.01 versus N-M617; ^○^ *P* < 0.05 versus DC.

In this experiment, we observed the impact of central GalR1 on plasma adiponectin levels in fat cells. As shown in Figure 3, the central administration of M617 elevated the adiponectin contents (*F*[3,32] = 124.7, *P* < 0.001). The adiponectin contents in the diabetic M617 group were enhanced by 7.2% (*P* < 0.05) compared with the diabetic controls but reduced by 22.7% (*P* < 0.01) compared with the nondiabetic M617 group. As compared with nondiabetic controls, the levels were increased by 5.5% (*P* < 0.05) in the nondiabetic M617 group but reduced by 23.9% (*P* < 0.01) in the diabetic control group. These results suggest that activation of GalR1 in brain may increase adiponectin release to ameliorate insulin resistance.

### 3.5. GLUT4 mRNA Expression Levels

**Figure 4:**
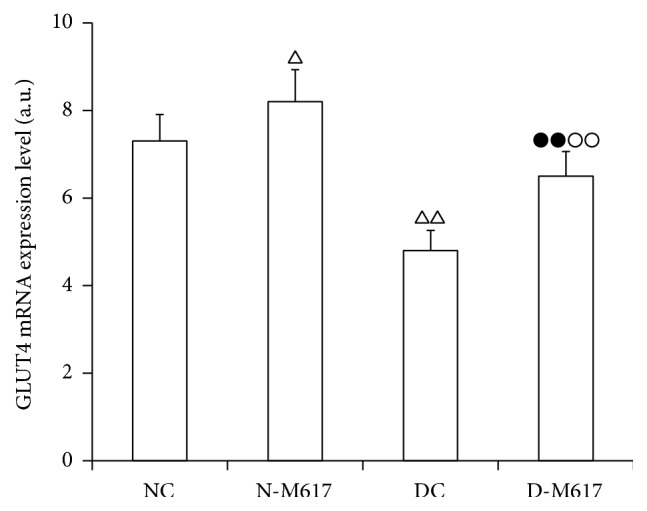
The i.c.v. injection of M617 significantly elevated GLUT4 mRNA expression levels in adipose cells (*n* = 8). The GLUT4 mRNA expression levels were higher in diabetic M617 (D-M617) and nondiabetic M617 (N-M617) groups as compared with diabetic controls (DC) and nondiabetic controls (NC), respectively. The expression levels were lower in D-M617 and DC groups compared with N-M617 and NC groups, respectively. The data shown are the means ± SEM. ^△^ *P* < 0.05, ^△△^ *P* < 0.01 versus NC; ^●●^ *P* < 0.01 versus N-M617; ^○○^ *P* < 0.01 versus DC.

In the present study, the central treatment with M617 significantly elevated the GLUT4 mRNA expression in fat cells of rats (*F*[3,32] = 65.7, *P* < 0.0001). As shown in Figure 4, the GLUT4 mRNA expression in the diabetic M617 group was increased by 36.3% (*P* < 0.01) compared with diabetic controls but reduced by 18.6% (*P* < 0.01) compared with the nondiabetic M617 group. As compared with nondiabetic controls, the GLUT4 gene expression was enhanced by 11.1% (*P* < 0.05) in the nondiabetic M617 group but significantly decreased by 33.7% (*P* < 0.01) in the diabetic control group.

### 3.6. GLUT4 Contents in Membranes of Fat Cells

**Figure 5:**
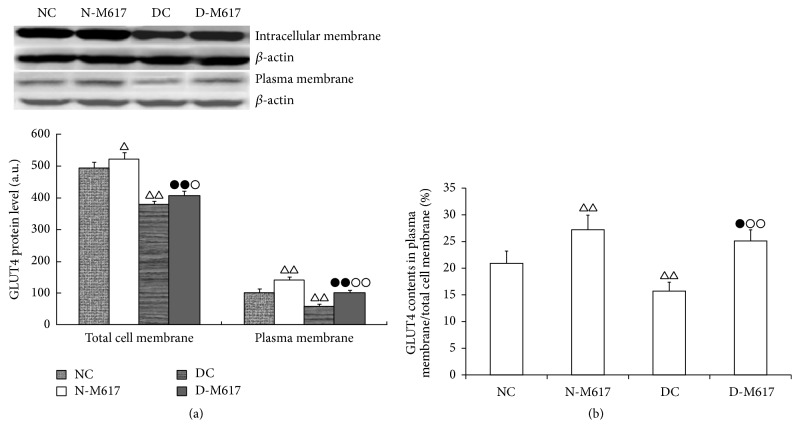
The i.c.v. administration of M617 enhanced GLUT4 contents and trafficking to plasma membranes of fat cells (*n* = 8). (a) The GLUT4 immunoreactivity in total cell membranes and plasma membranes was higher in diabetic M617 (D-M617) and nondiabetic M617 (N-M617) groups as compared with diabetic controls (DC) and nondiabetic controls (NC), respectively. The GLUT4 immunoreactivity in D-M617 and DC was lower as compared with N-M617 and NC, respectively, in total cell membranes and plasma membranes of adipose cells. (b) The central treatment with M617 enhanced the ratios of GLUT4 levels in plasma membranes to total cell membranes in D-M617 and N-M617 compared with each control, respectively. As compared with the diabetic controls, the ratio in N-M617 was increased, but in DC it was reduced. The data shown are the means ± SEM. ^△^ *P* < 0.05, ^△△^ *P* < 0.01 versus NC; ^●^ *P* < 0.05, ^●●^ *P* < 0.01 versus N-M617; ^○^ *P* < 0.05, ^○○^ *P* < 0.01 versus DC.

The central administration of M617 significantly elevated GLUT4 protein levels in both total cell membranes (*F*[3,32] = 108.3, *P* < 0.0001) and plasma membranes (*F*[3,32] = 132.8, *P* < 0.0001) of adipose cells (Figure 5(a)). The GLUT4 immunoreactivities in the diabetic M617 group were elevated by 7.2% (*P* < 0.05) in total cell membranes and 66.9% (*P* < 0.01) in plasma membranes compared with the diabetic controls but reduced by 22.2% (*P* < 0.01) in total cell membranes and 29.9% (*P* < 0.01) in plasma membranes compared with the nondiabetic M617 group. As compared with the nondiabetic control group the GLUT4 levels in the diabetic control group were reduced by 23.4% (*P* < 0.01) in total cell membranes and 42.2% (*P* < 0.01) in plasma membranes but were elevated by 5.6% (*P* < 0.05) in total cell membranes and 37.8% (*P* < 0.01) in plasma membranes in the nondiabetic M617 group.

Moreover, the central treatment with M617 significantly enhanced the ratios of GLUT4 levels in plasma membranes to total cell membranes (*F*[3, 32] = 77.3, *P* < 0.0001; Figure 5(b)). The ratios in the diabetic M617 group were enhanced by 56.1% (*P* < 0.01) compared with the diabetic control group but attenuated by 9.1% (*P* < 0.05) compared with the nondiabetic M617 group. As compared with nondiabetic controls, the ratios were elevated by 30.2% (*P* < 0.01) in the nondiabetic M617 group but reduced by 24.9% (*P* < 0.01) in the diabetic control group.

### 3.7. The Ratios of pAkt/Alt, pAS160/AS160, and pCREB/CREB

**Figure 6:**
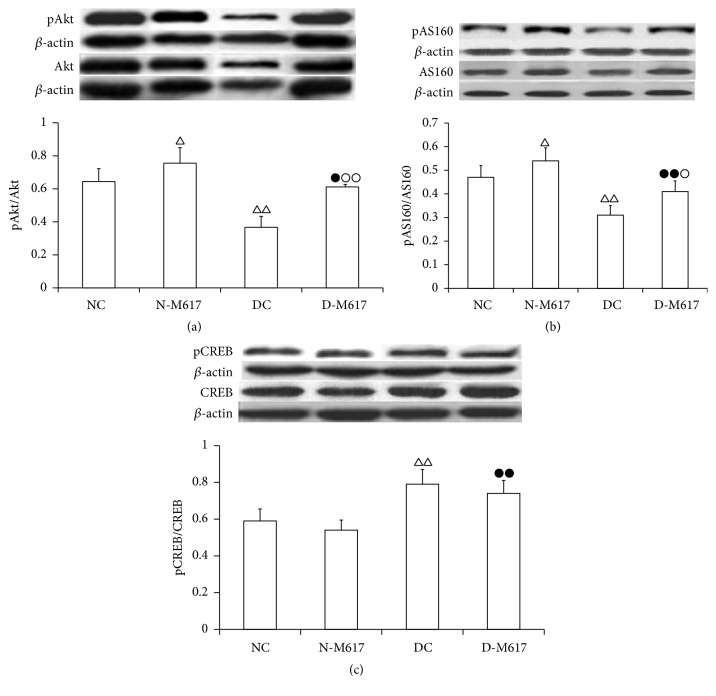
The i.c.v. injection of M617 significantly elevated ratios of pAkt/Akt and pAS160/AS160, but not ratios of pCREB/CREB in fat cells (*n* = 8). The ratios of pAkt/Akt (a) and pAS160/AS160 (b) were higher but not pCREB/CREB (c) in diabetic M617 group (D-M617) and nondiabetic M617 group (N-M617) than diabetic controls (DC) and nondiabetic controls (NC), respectively. The ratios of pAkt/Akt and pAS160/AS160 were lower, but pCREB/CREB were higher in D-M617 and DC than N-M617 and NC groups, respectively. All data shown are the means ± SEM. ^△^ *P* < 0.05, ^△△^ *P* < 0.01 versus NC; ^●^ *P* < 0.05, ^●●^ *P* < 0.01 versus N-M617; ^○^ *P* < 0.05, ^○○^ *P* < 0.01 versus DC.

As shown in Figure 6, the injection of M617 into the brain significantly elevated the ratios of pAkt/Akt (*F*[3, 32] = 10.8, *P* < 0.0001) and pAS160/AS160 (*F*[3, 32] = 18.9, *P* < 0.0001) but reduced the ratios of pCREB/CREB (*F*[3, 32] = 17.9, *P* < 0.0001) in adipose cells. The ratios of pAkt/Akt and pAS160/AS160 in the diabetic M617 group were enhanced by 70.9% (*P* < 0.01) and 31.8% (*P* < 0.05) compared with diabetic controls but decreased by 18.4% (*P* < 0.05) and 24.2% (*P* < 0.01) compared with the nondiabetic M617 group. The ratios of pCREB/CREB in the diabetic M617 group were increased by 36.1% (*P* < 0.01) compared with the nondiabetic M617 group, while they are not significantly altered (*P* > 0.05) compared with diabetic controls. As compared with nondiabetic controls, the ratios of pAkt/Akt and pAS160/AS160 in the diabetic control group were reduced by 43.1% (*P* < 0.01) and 33.9% (*P* < 0.01), but the ratios of pCREB/CREB were elevated by 34.6% (*P* < 0.01). Compared with nondiabetic controls, the ratios of pAkt/Akt and pAS160/AS160 in the nondiabetic M617 group were increased by 17.2% (*P* < 0.05) and 14.9% (*P* < 0.05), while the ratios of pCREB/CREB were not significantly altered (*P* > 0.05). These results suggest that activation of GalR1 in brain may increase pAkt and pAS160 contents, but not pCREB in adipose cells.

## 4. Discussion

Adipose tissue is considered not only as an energy storage depot, but also an important endocrine organ to regulate energy homeostasis by releasing adipokines, such as adiponectin [18, 19]. Although only 10% of insulin-stimulated glucose uptake takes place in the adipose tissue, it is very important to control whole-body energy homeostasis and glucose metabolism, as the GLUT4 expression level is selectively downregulated in adipose, not in skeletal muscle in insulin resistant states [20].

Several studies have implicated that activated GalR1 in brain is related to fat intake and body weight of subjects as galanin does. Reported initially by Dr. Lundström and colleagues, M617 can preferentially bind to GalR1 receptors and, therefore, is taken as a GalR1 preferring agonist [21]. The i.c.v. administration of M617, not GalR2 agonist, notably stimulated acute consumption of high-fat milk and cookie mash [22, 23]. In line with this, the results obtained in present study provided further evidences that activated GalR1 in the brain increased fat intake and body weight of rats, suggesting that GALR1 system at least partly mediated the galanin-induced appetitive effects. In addition, it is well known that during the compensated stage of type 2 diabetes, the circulatory insulin level of subjects is enhanced due to hyperglycemia stimuli and insulin resistance. After decompensation, the serum insulin level of subjects is reduced because of heavy damage of *β*-cells in the pancreas. Our data showed that the serum insulin levels were higher in the diabetic rats than controls, which might be blunted by central injection of M617, implicating that the diabetic rats used in this study were in the compensated period of pancreases and activated central GalR1 might inhibit insulin release as galanin did [24].

Adiponectin is a 30 kDa adipose-specific plasma protein. The plasma concentration of the protein in obese subjects and type 2 diabetic patients is decreased [25, 26]. Prospective longitudinal studies revealed that the plasma adiponectin levels declined at an early phase of obesity and further decreased after the development of type 2 diabetes [26]. Heterozygous adiponectin-deficient mice (adipo (+/−)) showed mild insulin resistance, while homozygous adiponectin-deficient mice (adipo (−/−)) suffered moderate insulin resistance with glucose intolerance [27]. It has been proved that a common silent T-G exchange in nucleotide 94 (exon 2) of the adiponectin gene associates with increased insulin resistance [28]. Intraperitoneal administration of 0.4 mg/kg globular adiponectin can improve insulin resistance and metabolic responses to insulin in male rats fed the high-fat diet via an AMPK- and nitric oxide-dependent mechanism [29]. In the current study the central administration of M617 elevated plasma adiponectin levels, suggesting that activated GalR1 in brain might stimulate adiponectin secretion from adipose tissues to benefit insulin sensitivity in diabetic rats.

Discovered from the plasma of patients with acute inflammation first, CRP is an independent risk factor for synthesizing macrophage inflammatory factors and developing insulin resistance [30]. CRP levels were reversely correlated with high-density lipoprotein contents but directly with plasma triglyceride and glucose concentrations [31]. Our current results indicated that the CRP levels were markedly decreased by central administration of M617, suggesting that activation of GalR1 in brain might reduce CRP discharge to ameliorate insulin resistance.

During the hyperinsulinemic-euglycemic clamp test, a constant insulin infusion increases glucose uptake into muscle and fat tissues and inhibits endogenous glucose production by the liver [10]. The amount of exogenous glucose required to maintain plasma glucose at its clamp level is quantified by the glucose infusion rate. Thus, an elevation of the glucose infusion rate in the clamp test indicates the increase in insulin sensitivity [8]. Our results in the clamp tests showed that the glucose infusion rat in the M617 group was higher than each control, suggesting that activation of GalR1 in brain might elevate the insulin sensitivity and benefit glucose clearance.

GLUT4 is responsible for insulin-stimulating glucose transport to keep blood glucose homeostasis [32]. Under a basal condition majority of GLUT4 is localized at intracellular membranes for its rapid endocytosis and slow exocytosis [33]. As stimulation of insulin, the recycle becomes rapid exocytosis and slow endocytosis, resulting in transporting more GLUT4 onto plasma membranes [34]. Only at plasma membranes can GLUT4 transport glucose into cells [8]. In addition, the GAL mRNA expressive level was directly correlated with GAL synthesis and release [10]. In the GAL-transgenic mice, the GalR1 mRNA expression was increased in discrete areas of the brain [15]. The present experimental results revealed that the central administration of M617 significantly upregulated GLUT4 protein and mRNA expression in adipose cells. Moreover, the ratios of GLUT4 contents in plasma membrane fractions to total cell membranes were higher in the M617 group than controls, implicating that injection of M617 not only enhances GLUT4 protein and mRNA expression, but also accelerates GLUT4 translocation from intracellular membranes to plasma membranes in adipose cells [7].

A number of studies have addressed the fact that GAL regulates glucose uptake and energy consumption at least via two signaling pathways in adipocytes. One is via activation of Gi/o receptors to inhibit adenyl cyclase and CREB [35]. The other is through upregulation of peroxisome proliferator-activated receptor protein and P2 gene expression to increase pAkt and pAS160 contents in adipocytes. Activation of Akt mediates the phosphorylation of AS160, which can promote hydrolysis of Rab-GTP to Rab-GDP to regulate GLUT4 translocation [36]. After administration of GAL, pAS160 inactivates GTPase and increases Rab-GTP activity to trigger GLUT4 translocation to plasma membranes to accelerate glucose uptake [37–39]. We found that central injection of M617 did not change ratios of pCREB/CREB but enhanced ratios of pAkt/Akt and pAS160/AS160 to boost GLUT4 translocation and glucose uptake, suggesting that activation of the Akt/AS160 pathway, not CREB, mediated the central GalR1 roles to increase glucose uptake.

Besides GalR1, M617 may also bind to GalR3 and activate the receptor [16]. However, the distribution of GAL receptor subtypes is species-specific. GalR1 and GalR2 are abundant in the brain of rat, whereas GalR3 is the important GAL receptor in the brain of human, especially in locus coeruleus of and dorsal raphe nucleus [39]. The ligand binding study revealed that GalR1 was the main GAL receptor subtype in the rodent brain [40]. In PVN of rat, GalR1 accounts for over 90% of all GAL binding sites [13]. The present findings showed that the beneficial effect of central M617 on insulin sensitivity was same as that of central GAL in adipose tissues [8–10], suggesting that the mitigatory effect of central GAL on insulin resistance is mainly mediated by activation of GalR1.

In brain, activation of GalR1 may be through three possible pathways to regulate insulin sensitivity. First, activation of GalR1 can release central transmitters, such as 5-HT [41, 42], NPY [43, 44], Ach [45], and NE [46] in different brain regions to regulate insulin sensitivity of subjects. Next, GAL-GalR1 system in brain can suppress sympathetic firing in a dose-dependent way, which is implicated in alleviation of insulin resistance [47, 48]. Consistent with this, GAL-knockout mice lack the sympathetic inhibition of insulin release [48]. Last, GAL-GalR1 system can alter the activities of central neurons in hypothalamus, amygdale, and arcuate nucleus to regulate energy homeostasis, which is relative to insulin sensitivity [49, 50]. The net effect of GalR1 deficiency was an impairment of glucose clearance following bolus glucose challenges [48]. Nonetheless, more studies are needed using GalR1 specific antagonists and GalR1 mutant mice to further explore the effect of central GalR1 on amelioration of insulin resistance.

In summary, the current results in the study suggest that activation of central GalR1 facilitates adiponectin release, glucose infusion rates in the hyperinsulinemic-euglycemic clamp test, GLUT4 mRNA expression, and GLUT4 translocation from intracellular membrane compartments to cell surfaces but inhibits the CRP release to benefit insulin sensitivity in adipose cells of type 2 diabetic rats. These effects may be partly due to triggering of the Akt/AS160 cascade, not CREB. This study contributes to our understanding of the central role of GalR1 in benefit of glucose uptake and insulin sensitivity, offering a possibility that the GalR1 may be taken as a potential therapeutic target for type 2 diabetes mellitus.

## Figures and Tables

**Table 1:** The effects of central injection of M617 on food intake, body weight, insulin, and plasma free fatty acid levels of rats (*n* = 8).

	Food intake (g/d)		Weight (g)		Insulin (mlU/L)		Free fatty acid (mmol/L)	
	Before	After	Before	After	Before	After	Before	After
NC	14.6 ± 1.0	15.2 ± 1.3	295.6 ± 11.2	302.4 ± 12.9	32.9 ± 4.7	33.1 ± 5.6	0.66 ± 0.6	0.69 ± 0.8
N-M617	14.8 ± 1.4	16.8 ± 1.2^△*∗∗*^	289.9 ± 11.4	316.3 ± 14.1^△*∗*^	34.5 ± 6.2	27.8 ± 0.5^△*∗∗*^	0.72 ± 0.7	0.64 ± 0.6^*∗*^
DC	15.7 ± 1.1^△^	16.6 ± 1.5^△^	260.1 ± 10.4^△△^	266.1 ± 11.2^△△^	37.9 ± 7.7^△^	40.4 ± 6.8^△^	1.15 ± 0.9^△△^	1.24 ± 1.1^△△^
D-M617	15.9 ± 1.6^●^	18.1 ± 1.3^●○*∗*^	255.2 ± 9.8^●●^	280.3 ± 12.7^●●○*∗∗*^	39.9 ± 7.9^●^	35.5 ± 6.6^●○*∗∗*^	1.19 ± 0.8^●●^	0.76 ± 0.8^●○○*∗∗*^
*F*[3, 32]	5.904	13.440	22.802	45.331	6.833	11.008	97.701	90.914
	*P* = 0.03	*P* = 0.0001	*P* = 0.0001	*P* = 0.0001	*P* = 0.001	*P* = 0.0001	*P* = 0.0001	*P* = 0.0001

^△^ *P* < 0.05, ^△△^ *P* < 0.01 versus nondiabetic control (NC) of each division; ^●^ *P* < 0.05, ^●●^ *P* < 0.01 versus N-M617 of each division; ^○^ *P* < 0.05,^ ○○^ *P* < 0.01 versus diabetic control (DC) of each division. ^*∗*^ *P* < 0.05, ^*∗∗*^ *P* < 0.01 versus those of before experiment of each division.

## Abbreviations

GAL: Galanin
GalR1: Galanin receptor 1
GLUT4: Glucose transporter 4
PVN: Paraventricular nucleus
i.c.v.: Intracerebroventricular
CREB: cAMP response element-binding protein
CRP: C-reactive protein
NPY: Neuropeptide Y.
